# EGAsubmitter: A software to automate submission of nucleic acid sequencing data to the European Genome-phenome Archive

**DOI:** 10.3389/fbinf.2023.1143014

**Published:** 2023-03-30

**Authors:** Marco Viviani, Marilisa Montemurro, Livio Trusolino, Andrea Bertotti, Gianvito Urgese, Elena Grassi

**Affiliations:** ^1^ Candiolo Cancer Institute—FPO IRCCS, Candiolo, Italy; ^2^ Department of Oncology, University of Torino, Candiolo, Italy; ^3^ Politecnico di Torino, Turin, Italy

**Keywords:** FAIR, EGA, raw data submission, automated workflows, DNA sequencing, metadata

## Abstract

Making raw data available to the research community is one of the pillars of Findability, Accessibility, Interoperability, and Reuse (FAIR) research. However, the submission of raw data to public databases still involves many manually operated procedures that are intrinsically time-consuming and error-prone, which raises potential reliability issues for both the data themselves and the ensuing metadata. For example, submitting sequencing data to the European Genome-phenome Archive (EGA) is estimated to take 1 month overall, and mainly relies on a web interface for metadata management that requires manual completion of forms and the upload of several comma separated values (CSV) files, which are not structured from a formal point of view. To tackle these limitations, here we present EGAsubmitter, a Snakemake-based pipeline that guides the user across all the submission steps, ranging from files encryption and upload, to metadata submission. EGASubmitter is expected to streamline the automated submission of sequencing data to EGA, minimizing user errors and ensuring higher end product fidelity.

## 1 Introduction

Recent technological advancements have made RNA and DNA sequencing a widely achievable task in both basic and translational research (Altman, 2012; Koboldt et al., 2013). The large body of sequencing data that is being generated is one of the driving forces behind the progressive rise of bioinformatics. On the one hand, an increasing amount of experiments now generate data that needs specific bioinformatics expertise to be analyzed. On the other hand, the progressive accumulation of new data creates opportunities to develop new approaches for data reanalysis and integration (Fasterius and Al-Khalili Szigyarto, 2018; Robertson et al., 2022). In this context of growing knowledge, it has become common practice that all the raw data obtained from a sequencing experiment are made available to the research community to ensure reproducibility and to provide usable information for follow-up studies (Baker, 2016).

Precise criteria on how we can address this need, among others, are defined by the FAIR principles for digital assets (Wilkinson et al., 2016), which posit that all the data and, at the same level of importance, metadata should be found and accessed by the community, emphasizing the need for machine actionability and shared dictionaries for metadata. When data is coming from human samples, FAIR principles are interpreted under the lens of protecting individual rights and privacy. Therefore, data repositories to store encrypted data and allow regulated access for research purposes only (as typically stated in the informed consent signed by patients or volunteers) have been developed and made available, such as the NCBI Sequence Read Archive (SRA (Leinonen et al., 2011)) and the European Genome-phenome Archive (EGA (Freeberg et al., 2022)). Many efforts are being directed towards developing reproducible methods and procedures, specifically for bioinformatics (Nüst et al., 2020; Papin et al., 2020), and the vast majority of journals now requires the raw data from all experiments to be deposited in one of the available repositories. However, much less work has been devoted to making data deposition efficient and error-free. There are proposals for shared solutions to define and share experimental metadata (González-Beltrán et al., 2014; Johnson et al., 2021), but right now different data repositories usually adopt their own ontologies and rules to create and manage metadata and a global consensus still has not been reached (Batista et al., 2022). We postulate that the same level of automation and minimal human intervention that is now required to run state-of-the-art analytical pipelines (Zhang and Jonassen, 2020) should be reached also in the context of data deposition. This would help researchers focus on the reliability and correctness of the metadata that they are submitting, rather than concentrating on the technicalities of the uploading procedure itself.

As a first step in this direction, we decided to develop EGA submissions, managing all the required procedures (files encryption and upload, and metadata linking and upload) with the specific aim of reducing human intervention to the least possible extent. The manual submission of a sequencing dataset to EGA is estimated to require 1 month overall (Submission FAQ - EGA European Genome-Phenome Archive, *“How long does a submission take?,”*) requiring researchers to go over a lengthy documentation in order to: 1) encrypt and upload all the data, 2) create several files to annotate them, and 3) work on a web interface for metadata linking and management. This course of action prompted different groups to implement their *ad hoc* solutions (Zhang, 2018; Band, 2019) to automate batch processing of multiple samples, with a number of unwanted consequences such as duplication of efforts, potential heterogeneity in metadata annotation, and limitations in the future interoperability of deposited data. Indeed, some public repositories with software aimed at supporting the interaction with EGA exist.

• star2xml (Barbero, 2022), a useful tool to ease the creation of an arduous file format like XML, which can be used for the EGA programmatic submission (Programmatic submissions (XML based)).
• EGA cluster cryptor (Kerssemakers and Strubel, 2020), which encrypts the files and uploads them to the user EGA box.
• EGA XML downloader (Kerssemakers, 2020), which allows the user to download European Nucleotide Archive (ENA) or EGA submissions.

Nevertheless, none of these software offers an integrated solution for the entire submission process, making EGAsubmitter, to our knowledge, the first tool that helps and guides users along all the steps to submit sequencing data to EGA. For EGAsubmitter implementation we adopted Snakemake, a well-known pipeline management system (Köster and Rahmann, 2012) applicable to make new features easy to develop for computational biologists and to offer, at the same time, an easy-to-use tool for researchers without specific bioinformatics knowledge.

## 2 Methods

**FIGURE 1:**
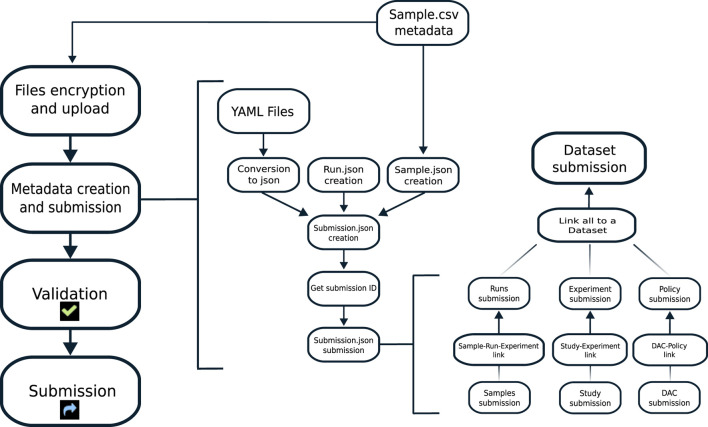
Workflow of the pipeline. Left: the main steps are shown. The tool encrypts and uploads to the EGA box all the files listed by the user. Middle: starting from YAML files and the CSV file filled in by the user, EGA entities are created. Right: all the needed entities are linked, following the required data model, and submitted. At the end of the process, entities are validated by EGA and the submission process is completed.

The tool we are presenting is based on the EGA tool EGACryptor to encrypt the files and on python scripts for files' upload and metadata files’ creation. All the steps are linked using a Snakemake-based pipeline, to ease the automation of all the process (Figure 1).

Through this, we were able to reduce the needed input files to one main comma separated values (CSV) file and five YAML type files, the latter corresponding to each of the entities that EGA requires for a submission.

• **Study**, a brief description about the sequencing study that is being submitted.
• **Experiments**, which are information about the sequencing methods, protocols and machines used for the presented data. Experiments generate the linkage between samples and study and are necessary for FASTQ and BAM/CRAM submissions only.
• **Data Access Committee** (DAC), information about the person who will be responsible for giving access to third parties to download the data.
• **Policy**, which contains the rules on how and by whom the submitted data can be used; these are usually defined in the consent signed by patients and are reported in the Data Access Agreement (DAA) signed while creating the account. Policy is linked with the DAC.
• **Dataset**: contains the collection of Runs data files to be subject to controlled access. It is linked with the Policy.

Moreover, EGAsubmitter automatically creates two other important entities, **Samples** and **Runs**, one for each sample. ‘Samples’ entities are created starting from the CSV file and include all descriptions and metadata filled in by the user; ‘Runs’ entities are created to link Samples, Experiments and Files together. As an example, for the File “file_R1.fastq.gz,” the Sample entity identifies all the metadata of the sample that originated the data analyzed through the RNAseq experiment, which is detailed in the Experiment entity. The relationship between these three entities (File, Sample, and Experiment) is represented with a fourth entity, called Run, which links together the results of a given Experiment on a specific Sample, whose corresponding data are stored in the specified FASTQ file.

The CSV needs to be filled in with all the available sample metadata, as requested by EGA, plus two other pieces of information: the name of the file that the user intends to upload, and the local path where it is stored. YAML files should be filled in with all the information available to the user about the experiment that generated the data.

After preparing these files, the user can launch the first part of the process where all the files are encrypted using the tool given by EGA itself (EGACryptor), which also creates both unencrypted and encrypted MD5 checksum for each file. Those checksums are used by EGA, after datasets submission, to ensure that no data corruption occurred during the upload process. Upon completion of this step, EGAsubmitter automatically starts to upload all the files through File Transfer Protocol (FTP).

Thereafter, the user can start to upload samples metadata: in this step, EGAsubmitter converts all YAML files to JSON type files, and creates one JSON file for each sample of the CSV file, containing all the needed information. Moreover, the tool creates one JSON for the Runs entities, where the sample alias, the associated file name and its previously created unencrypted and crypted MD5 checksum are automatically stored.

After this, thanks to sequential Snakemake rules, the tool links together entities that need to be linked, and uploads everything to the EGA database. The link is generated by obtaining the ID given by EGA to each entity once it is uploaded and by adding the newly retrieved ID to the JSON of the related entities.

The pipeline steps described here can be launched separately thanks to different bash scripts. In any moment, the user can decide to abort the submission, if some errors in the metadata or list of files have been detected, repeating only the encryption and upload phase and/or the submission of the metadata. EGAsubmitter will notify the user if there are errors that can be detected automatically, for example, a submission that began for paired end data without paired FASTQ files for all samples.

Once everything is uploaded, the pipeline stops. At this time, the user can check the status of the submission directly on the EGA Submitter Portal, continue with the validation process and, if successful, finalize the submission. If anything happens during pipeline execution (for example, if a network error occurs), the tool is designed to (re)start where the process was interrupted, without repeating any step.

Lastly, when the submission is finalized (blue S on the EGA portal), EGA assigns a specific ID to each entity, identified by the name “EGA” plus a letter, specific for each object (e.g., EGAS: for the Study Accession ID, EGAC for DAC Accession ID, etc., visit the EGA submitter portal, “Identifier” for details). This information can be listed in manuscripts submitted for consideration with a link to the EGA submission, to ease reproducibility. EGAsubmitter can retrieve the EGA IDs, building a final TSV file listing IDs of each Sample and Run, as well as those of the DAC, Experiment, Dataset and Policy.

All the required dependencies can be easily installed using conda as a package management system, to make it easily portable and reproducible. Detailed instructions about the whole process and a representative example of the CSV file can be found in the Supplementary Text S1, the source code repository README and Supplementary Table S1.

## 3 Results

According to EGA “We […] suggest to anticipate that the submission process will take at least 1 month” (Submission FAQ - EGA European Genome-Phenome Archive, “*How long does a submission take?*”). Considering a small/medium dataset (about 200–400 samples), the time required for a full submission using EGAsubmitter is reduced to about 2 hours of human work, all focused on defining the metadata and then letting the encryption-upload process proceed on its own.

The software is designed to restart the task from partially uploaded files if needed, allowing the user to concentrate efforts on curating the metadata. This part can be done using a given set of precompiled structured and intuitive YAML files, which the user is asked to fill in, and only a single CSV file, listing all the samples and incorporating specific information about them (e.g., sample ID, disease type, patient gender, etc.). Linking between all the EGA entities is automatically managed by the different rules of the pipeline.

### 3.1 Execution times of a real submission

We used EGAsubmitter for some of our real-life submissions. Considering a dataset of 119 samples with an average FASTQ size of 3.1Gb, on a standard linux system, with Intel Xeon Gold 6252 (3.700 GHz) as cores, we registered times of: 20–30 min for encryption (10 cores selected, maximum RAM usage 1.6 Gb), 1 h for upload, and 10–20 min for metadata link and submission.

Clearly, these time recordings are heavily dependent on the bandwidth available for the upload and the available cores for the encryption phase.

The 2 hours of human work that we anticipate, apart from creating the CSV and the YAML files, are mainly related to all the steps that require a direct interaction with EGA: to start the process users will need to write an e-mail to obtain credentials to access the FTP and the EGA Submitter Portal. Afterwards, when they are done with EGAsubmitter, they will proceed with finalization on the Submitter Portal. Finally they should contact the helpdesk again to ask the release of the Study.

## 4 Discussion

We offer a tool, EGAsubmitter, to automatically upload data to the European Genome-phenome Archive. This tool will hopefully reduce errors and the time needed to troubleshoot submissions and is particularly meant to help those who are not very familiar with these kinds of applications. In the current version, EGAsubmitter is able to manage both single- and paired-end type FASTQ (Cock et al., 2010) files and BAM (Li et al., 2009), with the possibility to upload both BAM and the derived FASTQ in the same submission instance. The packages required for the tool to work properly are loaded *via* a conda environment. Moreover, EGAsubmitter can be used to upload CRAM files or other less common file types for deep sequencing projects, such as FASTA, Standard Flowgram Files (SFF), and Sequence Read Format (SRF).

Notably, the user will still need to check the submission progress and validate and request the final submission of metadata by themselves, using the EGA Submitter Portal. We are convinced that a last check on the portal, before proceeding with the validation and submission of the entire study to EGA, represents a safe and efficient middle ground between complete automation and human interventions, which we have deliberately kept at a minimum while cautiously maintaining a manual intervention at the beginning and at the end of the process.

When developing EGAsubmitter, our design choices regarding user input aimed at keeping them as simple as possible, specifically we followed the metadata structure that is required by EGA and their specific dictionaries for different entries. For this reason we used YAML and a single CSV and we did not implement a dedicated layer to manage metadata adopting a specific library for it (Chalk, 2016; Batista et al., 2022); future developments would definitely focus on integrating EGA requirements with an existing metadata model, striving to keep the user interface as easy as possible but at the same time reaching better interoperability and quality of annotations.

As it is now, EGAsubmitter is designed to make submissions to EGA as effortless as possible, but it also represents a starting point to automate submissions to other repositories, such as GEO or ENA. Harmonizing metadata definitions across repositories is a fundamental step to make datasets easier for publication and interrogation, more usable by the research community, and more endowed with interoperable annotations.

## Data Availability

The original contributions presented in the study are included in the article/Supplementary Material, further inquiries can be directed to the corresponding author. (https://github.com/bioinformaticspolito/EGAsubmitter.git)

## References

[B1] Altman R. B. (2012). Translational bioinformatics: Linking the molecular world to the clinical world. Clin. Pharmacol. Ther. 91, 994–1000. 10.1038/clpt.2012.49 22549287 PMC4154360

[B2] Baker M. (2016). 1,500 scientists lift the lid on reproducibility. Nature 533, 452–454. 10.1038/533452a 27225100

[B3] Band G. (2019). Me vs. EGA. Available at: https://gavinband.github.io/bioinformatics/data/2019/05/01/Me_versus_the_European_Genome_Phenome_Archive.html .

[B4] Barbero M. C. (2022). Star2xml. Available at: https://github.com/EGA-archive/star2xml .

[B5] Batista D. Gonzalez-Beltran A. Sansone S.-A. Rocca-Serra P. (2022). Machine actionable metadata models. Sci. data 9, 592. 10.1038/s41597-022-01707-6 36180441 PMC9525592

[B6] Chalk S. J. (2016). SciData: A data model and ontology for semantic representation of scientific data. J. cheminformatics 8, 54. 10.1186/s13321-016-0168-9 PMC5064921 27795738

[B7] Cock P. J. Fields C. J. Goto N. Heuer M. L. Rice P. M. (2010). The sanger fastq file format for sequences with quality scores, and the solexa/illumina fastq variants. Nucleic Acids Res. 38, 1767–1771. 10.1093/nar/gkp1137 20015970 PMC2847217

[B8] Fasterius E. Al-Khalili Szigyarto C. (2018). Analysis of public RNA-sequencing data reveals biological consequences of genetic heterogeneity in cell line populations. Sci. Rep. 8, 11226. 10.1038/s41598-018-29506-3 30046134 PMC6060100

[B9] Freeberg M. A. Fromont L. A. D’Altri T. Romero A. F. Ciges J. I. Jene A. (2022). The European genome-phenome archive in 2021. Nucleic Acids Res. 50, D980–D987. 10.1093/nar/gkab1059 34791407 PMC8728218

[B10] González-Beltrán A. Neumann S. Maguire E. Sansone S.-A. Rocca-Serra P. (2014). The risa r/bioconductor package: Integrative data analysis from experimental metadata and back again. BMC Bioinforma. 15 (1), S11. 10.1186/1471-2105-15-S1-S11 PMC4015122 24564732

[B11] Johnson D. Batista D. Cochrane K. Davey R. P. Etuk A. Gonzalez-Beltran A. (2021). ISA API: An open platform for interoperable life science experimental metadata. GigaScience 10, giab060. 10.1093/gigascience/giab060 34528664 PMC8444265

[B12] Kerssemakers J. (2020). EGA XML downloader. Available at: https://github.com/DKFZ-ODCF/ega-xml-dl .

[B13] Kerssemakers J. Strubel P. (2020). EGA cluster cryptor. Available at: https://github.com/DKFZ-ODCF/ega-cluster-cryptor .

[B14] Koboldt D. C. Steinberg K. M. Larson D. E. Wilson R. K. Mardis E. R. (2013). The next-generation sequencing revolution and its impact on genomics. Cell. 155, 27–38. 10.1016/j.cell.2013.09.006 24074859 PMC3969849

[B15] Köster J. Rahmann S. (2012). Snakemake–a scalable bioinformatics workflow engine. Bioinformatics 28, 2520–2522. 10.1093/bioinformatics/bts480 22908215

[B16] Leinonen R. Sugawara H. Shumway M. Collaboration I. N. S. D. (2011). The sequence read archive. Nucleic Acids Res. 39, D19–D21. 10.1093/nar/gkq1019 21062823 PMC3013647

[B17] Li H. Handsaker B. Wysoker A. Fennell T. Ruan J. Homer N. (2009). The sequence alignment/map format and samtools. Bioinformatics 25, 2078–2079. 10.1093/bioinformatics/btp352 19505943 PMC2723002

[B18] Nüst D. Sochat V. Marwick B. Eglen S. J. Head T. Hirst T. (2020). Ten simple rules for writing Dockerfiles for reproducible data science. PLoS Comput. Biol. 16, e1008316. 10.1371/journal.pcbi.1008316 33170857 PMC7654784

[B19] Papin J. A. Mac Gabhann F. Sauro H. M. Nickerson D. Rampadarath A. (2020). Improving reproducibility in computational biology research. PLoS Comput. Biol. 16, e1007881. 10.1371/journal.pcbi.1007881 32427998 PMC7236972

[B20] Robertson A. J. Tan N. B. Spurdle A. B. Metke-Jimenez A. Sullivan C. Waddell N. (2022). Re-analysis of genomic data: An overview of the mechanisms and complexities of clinical adoption. Genet. Med. 24, 798–810. 10.1016/j.gim.2021.12.011 35065883

[B21] Wilkinson M. D. Dumontier M. Aalbersberg I. J. J. Appleton G. Axton M. Baak A. (2016). The FAIR guiding principles for scientific data management and stewardship. Sci. data 3, 160018. 10.1038/sdata.2016.18 26978244 PMC4792175

[B22] Zhang J. (2018). EGASUB - ICGC EGA submission CLI, Available at: https://github.com/icgc-dcc/egasub

[B23] Zhang X. Jonassen I. (2020). RASflow: An RNA-seq analysis workflow with Snakemake. BMC Bioinforma. 21, 110. 10.1186/s12859-020-3433-x PMC7079470 32183729

