# Supplementary material for: EGAsubmitter: A software to automate submission of nucleic acid sequencing data to the European Genome-phenome Archive
Source: Front Bioinform. 2023 Mar 30;3:1143014. doi: 10.3389/fbinf.2023.1143014 (PMC10098081; doi:10.3389/fbinf.2023.1143014)
Supplement: Supplementary file 1 [file Presentation1.pdf]

Supplementary Material

1 SUPPLEMENTARY DATA

Let's imagine we have five lung samples named Sample_RTV.ABCXYZ, Sample_GYH.DEFXYZ, Sample_a.SAS.GHIXYZ, Sample_b.SAS.GHIXYZ, and Sample_QWE.YKLXYZ, which have been analyzed with RNA sequencing with single-end reads: therefore, we will need to upload one fastq file for each sample. Usually, fastq files are named "_R1", and "_R2" for the two read ends; therefore we should have Sample_RTV.ABCXYZ_R1_fastq.gz, Sample_GYH.DEFXYZ_R1_fastq.gz, Sample_a.SAS.GHIXYZ_R1_fastq.gz, Sample_b.SAS.GHIXYZ_R1_fastq.gz, and Sample_QWE.YKLXYZ_R1_fastq.gz.

To start, we clone the EGAsubmitter repository with `$ git clone https://github.com/bioinformatics-polito/EGAsubmitter.git` on the computer where files are stored. Then we move inside the new folder, where we will find, among the others, a file named "EGAsubmitter.yml": this is the environment to install with conda in order to have all the needed packages.
We can create a new conda environment starting from this file using `$ conda env create -f EGAsubmitter.yml`. Then we can activate it with `$ conda activate EGAsubmitter`. The repository contains all the files the user will need to fill in as templates. The CSV with samples and their related files information consists of 20 columns. Considering our four samples written above, the CSV files should look like Supplementary Table 1.
NOTE: for each sample there should be exactly one file, being a FASTQ (two FASTQ if you have paired sequence) or a BAM, or whatever type you are submitting. If you plan to submit only one file type, even if it is a BAM, always use "fileName" and "filePath" columns. The other two columns, named "fileName.bam" and "filePath.bam", must be used **only** if you plan to submit FASTQ and BAM together.

The next step will extract the information needed for the encryption and upload of files, which are the alias of the sample, the file name associated with that sample and the path where this file is stored locally. We can use the command `$ ./getPaths.sh` and the file will be created using the information we put in the CSV file. Now, we can login using the command `$ source ./login.sh` and insert our EGA credentials. After this, we are ready to proceed to the encryption and upload of our files, with the command `$ ./encrypt-upload.sh`. We will be asked for a folder name to identify the project and where to store all the encrypted files, and the number of cores to use. The tool will get all the files from paths the user has specified, encrypt and upload them, using the specified number of cores.

During encryption and upload we can fill in all the YAML files. Every YAML is a template and contains comments on what needs to be written in it. Here is an example: this is Study.yaml.

```
alias: '' # Do not fill
studyTypeId: '' # local/share/data/metadata/enums/studyTypeId.txt
shortName: '' # Not required
title: ''  # The title of your work: same for every "title"
studyAbstract: '' # The abstract of the paper (if present already), or a short summary
ownTerm: '' # Not required
pubMedIds: [] # Not required
customTags: # Not required
```

```
    - tag: ''
    - value: ''
```

The “studyTypeId” entry is noteworthy: to fill it properly, the user should look for the value which better describes the study that is going to be uploaded, in the corresponding file, as suggested by the comment, present in
`local/share/data/metadata/enums`. The said file is “studyTypeId.txt” (A markdown version of these files is also available for readability):

```
tag value
0   Whole Genome Sequencing
1   Metagenomics
2   Transcriptome Analysis
3   Resequencing
4   Epigenetics
5   Synthetic Genomics
6   Forensic or Paleo-genomics
7   Gene Regulation Study
8   Cancer Genomics
9   Population Genomics
10  RNASeq
11  Exome Sequencing
12  Pooled Clone Sequencing
13  Transcriptome Sequencing
14  Other
```

In this guide, we are trying to upload a RNAseq project, so we write 10 in the “studyTypeId” line. The same procedure will need to be followed for all the YAML fields where an ”association list” is mentioned in comments.
Once all the YAML files have been filled in, and the encrypt-upload process has ended, we can launch the command `$ ./metadataSubmission.sh`. This will take care of the biggest part of the submission process to EGA, storing every information in JSON files that then will be uploaded.
Once everything has ended, we can go on EGA Submitter Portal, login, and check our new submission. If everything is ok, we can just click on the green check “Validate” in order to start the submission validation. If the validation has been successful, the yellow D (“Draft”) on the left will change to a green V (“Validated”) and we can proceed with the submission clicking on the light blue arrow (“Submit”).

WARNING: There exists a time window between the data upload and the availability of such files via the Submitter Portal. For this reason we suggest to proceed to the validation the day after uploading the files. Submission FAQ - EGA European Genome-Phenome Archive, *”Why my files are not available if I see them in the FTP box?”*. If the validation or the submission is not completely done, we would see a red VE (“Validated with errors”) or a SP (“Submitted Partially”) and we will need to look at the Submission errors console inside our submission to find out what went wrong. Once we have solved everything, we can try the validation or the submission again. The whole process is now complete, and you can write an email to the EGA helpdesk (ega-helpdesk@ebi.ac.uk) to publish your dataset.
